# Primary immunodeficiency disease: a retrospective study of 112 Chinese children in a single tertiary care center

**DOI:** 10.1186/s12887-019-1729-7

**Published:** 2019-11-04

**Authors:** Jinhong Wu, Wenwei Zhong, Yong Yin, Hao Zhang

**Affiliations:** Department of Pulmonary, Shanghai Children’s Medical Center Affiliated to Shanghai Jiaotong University School of Medicine, Shanghai, 200127 China

**Keywords:** Primary immunodeficiency disease, Clinical features, Demographic characteristics, Children, China

## Abstract

**Background:**

Primary immunodeficiency disease (PID) is a disorder caused by an inherited flaw in the immune system that increases the susceptibility to infections.

**Methods:**

In this study, 112 children with PID were diagnosed and classified based on the 2017 criteria presented by the International Union of Immunological Societies (IUIC) in a single tertiary care center from January 2013 to November 2018. We retrospectively studied the clinical features of those PID children and followed-up them as well.

**Results:**

It was revealed that male/female ratio was 6:1. The most frequent diagnosed PID was severe combined immunodeficiency (SCID) (28.6%) and hyper-IgM (HIGM) syndrome (24.1%), followed by predominantly antibody deficiencies (17.8%). Combined immunodeficiencies with associated or syndromic features (12.5%) and congenital defects of phagocyte number, function, or both (10.7%) were less common in our center compared with SCID and HIGM syndrome. Besides, we found that 20 children (17.8%) had a positive family history of PID, and almost all cases (97.3%) had a history of recurrent infection. Recurrent respiratory tract infection was among the most common symptoms, followed by the bacterial infection of the skin and mucous membranes and diarrhea. Additionally, adverse event following immunization (AEFI) was found in 20.5% of the patients, and immune disorder was commonly observed in PID patients. In the present study, 47 patients underwent allogeneic hematopoietic stem cell transplantation (allo-HSCT), and 2-year overall survival (OS) rate for these patients was 78.7% (37/47). It is noteworthy that OS widely differed among PID patients with different phenotypes who underwent allo-HSCT. The 2-year OS rate for SCID, HIGM syndrome, and the remaining of PID patients who underwent allo-HSCT was 14.3, 83.3, and 100%, respectively.

**Conclusions:**

PID typically emerges at early age. Recurrent infection and serious infection were the most common clinical manifestations. Allo-HSCT is a relatively effective therapeutic strategy for PID patients.

## Background

Primary immunodeficiency disease (PID) refers to a heterogeneous group of over 130 disorders that result from defects in immune system development and/or function. With the development and use of flow cytometry and genetic technology for clinical diagnosis, diagnostic rate of PID has been markedly increased in China. To date, 354 distinct disorders with 344 different gene defects have been identified in patients with PID [1]. PID is characterized by diverse clinical manifestations, such as recurrent or prolonged serious infections, autoimmune/inflammatory disease, allergy, or malignancy. In addition, physicians or general practitioners may not able to recognize PID because of rarity of those diseases, particularly in developing countries (e.g., China) [2, 3]. As a result, delayed diagnosis or misdiagnosis are quite common in clinical practice, which may lead to the poor outcomes for children with PID. In the present study, we retrospectively analyzed the clinical features and prognosis of 112 PID children over a 6-year period in a single tertiary care center to strengthen our understanding about PID.

## Methods

### Classification and diagnosis

A single-center retrospective study was carried out at Shanghai Children’s Medical Center Affiliated to Shanghai Jiao Tong University (Shanghai, China) from January 2013 to November 2018. A total of 112 patients were included for investigation and classified according to the 2017 criteria presented by the International Union of Immunological Societies (IUIS) classification system [1]. In addition, PID was grouped as follows: immunodeficiencies affecting cellular and humoral immunity; predominantly antibody-deficiency diseases; combined immunodeficiencies with associated or syndromic features; diseases of immune dysregulation; congenital defects of phagocyte number or function; defects in intrinsic and innate immunity; autoinflammatory disorders; and complement deficiencies [4, 5]. Because the clinical features and prognosis of hyper-IgM (HIGM) syndrome are different from those associated with severe combined immunodeficiency (SCID), patients with HIGM syndrome were not herein included in the group of immunodeficiencies influencing cellular and humoral immunity. All patients were diagnosed according to the diagnostic criteria and confirmed by genetic phenotyping. Immunodeficiencies secondary to other conditions (e.g., human immunodeficiency virus (HIV) infection) were excluded as well.

### Data collection

All clinical data were collected from the hospital medical records. Sex, family history, age of the first hospitalization, clinical features, onset of symptoms, signs, age of onset, age at diagnosis, auxiliary examination, and disease progress were included in this study.

### Specimen collection and sequencing analysis of relevant genes

Patients’ specimens were collected during hospitalization from January 2013 to November 2018. For comparative purposes, we also collected specimens from the parents. Anticoagulant venous blood was collected and was further centrifuged at 3500 rpm for 10 min. Peripheral blood cells were stored at − 80 °C and used for DNA extraction and gene sequencing using next-generation sequencing (NGS) technique, which were further performed based on the clinical features and auxiliary examination results.

### Statistical analysis

Data were analyzed by using SPSS 17.0 software (IBM, Armonk, NY, USA). The differences between the groups were compared by the Student’s *t*-test. *P* values < 0.05 were considered statistically significant.

## Results

### Demographic features of PID patients

#### Frequency and distribution of PID

Diagnosis was based on clinical, immunological, and genetic testing, and 112 patients were diagnosed with PID in our hospital from January 2013 to November 2018. There were 18 PIDs diagnosised in 112 patients with pathogenic mutations which grouped into 7 main categories (Table 1). As shown in Table 1, SCID was found in 32 patients (28.6%), HIGM syndrome in 27 (24.1%), combined immunodeficiencies with associated or syndromic features 14 (12.5%), predominantly antibody deficiencies in 20 (17.8%), diseases related to immune dysregulation in 4 (3.6%), congenital defects of phagocyte number or function, or both in 12 (10.7%), defects in intrinsic and innate immunity in 2(1.8%), and complement deficiencies in 1 (0.9%) (Table 1). None of the patients were diagnosed with phenocopies of PID or defects in autoinflammatory disorders. Combination of T- and B-cell immunodeficiencies was the most common (*n* = 32) among all the patients with PID, followed by HIGM syndrome (*n* = 27). Wiskott–Aldrich syndrome (WAS) was the most common PID in combined immunodeficiencies with associated or syndromic features group (*n* = 9), followed by dyskeratosis congenita (*n* = 2). Predominantly antibody deficiencies were the most common PIDs in patients with severe serum immunologlobulin isotypes reduction and B cells decreased or absent(*n* = 16), followed by common variable immunodeficiency disorders (*n* = 3). There were 7 and 5 patients who diagnosed with chronic granulomatous disease and congenital neutropenias, respectively. Those patients showed congenital defects in phagocyte number, function or both.

**Table 1:** Distribution of PIDs  according to the International Union of Immunological Societies criteria

Type	No. of Cases (%)
Immunodeficiencies affecting cellular and humoral immunity	59 (28.6)
SCID	32
HIGM	27
Combined immunodeficiencies with associated or syndromic features	14 (12.5)
WAS	9
DKC	2
DGS	1
EDA-ID	1
HIES	1
Predominantly antibody deficiencies	20 (17.8)
Severe reduction in all serum immunologlobulin isotypes with profoundly decreased or absent B cells	16
CVID	3
APDS	1
Diseases of immune dysregulation	4 (3.6)
IPEX syndrome	2
Immune dysregulation with colitis	1
CHS	1
Congenital defects of phagocyte number or function	12 (10.7)
CGD	7
Congenital neutropenias	5
Defects in intrinsic and innate immunity	2 (1.8)
IFN-γ receptor deficiency	1
Predisposition to invasive fungal diseases (CARD9 deficiency)	1
Complement deficiencies	1 (0.9)
Complement deficiencies	1

Abbreviations: *APDS* activated PI3Kδ syndrome immunodeficiency, *CGD* chronic granulomatous disease, *CHS* Chediak-Higashi syndrome, *CVID* common variable immunodeficiency disorders, *DGS* DiGeorge syndrome, *DKC* dyskeratosis congenital, *EDA-ID* anhidrotic ectodermodysplasia with immunodeficiency, *HIGM* hyper-IgM syndrome, *HIES* Hyper-IgE syndromes, *IPEX* immunodysregulation, polyendocrinopathy, enteropathy X-linked syndrome, *SCID* severe combined immune deficiency, *WAS* Wiskott–Aldrich syndrome, *XLA* X-linked agammaglobulinemia

#### Patients’ demographic characteristics

There were 96 males and 16 females in the present study. The male/female ratio was 6:1, and there were different ratios among subgroups of PIDs (Table 2). All patients with PID related to X-linked recessive inheritance were male (e.g., Bruton tyrosine kinase (BTK)-deficiency, WAS, and HIGM syndrome).

**Table 2:** General characteristics of patients with primary immunodeficiency diseases

Category	No. of Cases	Gender Ratio (F/M)	No. of Death	Age at death (mo)	No. of HSCT	Age at onset (mo)	Age at diagnosis (mo)	Family history
SCID	32	22/10	30	7.03 ± 1.25 (1-28)	7	4.03 ± 0.88 (0.5-23)	5.94 ± 1.06 (1-28)	7
HIGM	27	27/0	4	88.50 ± 44.66 (16-205)	24	9.536 ± 2.23 (1-60)	31.25 ± 6.23 (3-120)	4
WAS	9	9/0	0		9	1.00 ± 0.14 (0.5-2)	7.22 ± 1.98 (2-22)	0
DGS	1	1/0				0.5	0.5	0
DKC	2	2/0	1	26	1	37.00 ± 23.00	44.00 ± 20.00	1
EDA-ID	1	1/0	1	4		0.5	3	1
HIES	1	1/0	1	133		60	60	0
Severe reduction in all serum immunologlobulin isotypes with profoundly decreased or absent B cells	16	15/1	3	46.00 ± 23.29 (3-83)		26.50 ± 6.89 (2-60)	41.80 ± 10.44 (3-120)	3
CVID	3	3/0	0			29.33 ± 13.13 (4-48)	46.33 ± 26.21 (7-96)	0
APDS	1	1/0	0			96	96	0
IPEX syndrome	2	2/0	0		2	6, 28	6, 28	0
Immune dysregulation with colitis	1	0/1	1	13		1	4	0
CHS	1	0/1	0		1	2	68	0
Congenital neutropenias	5	4/1	0		1			2
CGD	7	6/1	1	12	1	2.17 ± 1.17 (1-8)	5.00 ± 2.11 (1-15)	2
IFN-γ receptor deficiency	1	0/1	0		1	2	60	0
Predisposition to invasive fungal diseases (CARD9 deficiency)	1	1/0	0			117	130	0
Complement deficiencies	1	1/0	0			48	60	0

Abbreviations: *APDS* activated PI3Kδ syndrome immunodeficiency, *CGD* chronic granulomatous disease, *CHS* Chediak-Higashi syndrome, *CVID* common variable immunodeficiency disorders, *DGS* DiGeorge syndrome, *DKC* dyskeratosis congenital, *EDA-ID* anhidrotic ectodermodysplasia with immunodeficiency, *HIGM* hyper-IgM syndrome, *HIES* Hyper-IgE syndromes, *IPEX* immunodysregulation, polyendocrinopathy, enteropathy X-linked syndrome, *SCID* severe combined immune deficiency, *WAS* Wiskott–Aldrich syndrome, *XLA* X-linked agammaglobulinemia

#### Distribution of age in patients with PID

The average onset age of all patients with PID was 13 months (range, 2 days-117 months). The onset age of 68 children (60.7%) was younger than 6 months, there were 28 (25%) patients aged between 6 months and 3 years, and 16 (14.3%) patients were older than 3 years as well. The average time to the first diagnosis as PID was 24 months (range, 1-130 months). Besides, 38 (33.9%) children were diagnosed with PID who were younger than 6 months, 45 (40.2%) children aged between 6 months and 3 years, and 29 (25.9%) children were older than 3 years (Table 2). There were statistically significant difference among subgroups of PID according to the age of onset and age at the time of diagnosis (Table 2). Our study indicated that symptoms were notably observed earlier in WAS and SCID patients compared with other subgroups with the median onset age of 1 and 4 months, respectively; meanwhile, the time of the first diagnosis was shorter in WAS and SCID patients with the median diagnosis time of 7.22 and 5.94 months, respectively. Compared with WAS and SCID patients, patients with primary antibody deficiency displayed symptoms remarkably later with the median age of 26.5 months, in which the mean time to diagnosis was 41.8 months.

#### Main clinical manifestations

In our study population, 20 patients (17.8%) had a positive family history of PID (Table 2). Additionally, patients with PID presented various clinical manifestations during diagnosis (Table 3). A previous history of recurrent infection was found in almost all the patients (*n* = 109, 97.3%). The main pathogen of infection was bacterial infection (*n* = 108), and fungal infection (*n* = 18) ranked in the second place, followed by viral infection in the third place (*n* = 7). Respiratory infection was the most common complication (*n* = 89, 79.5%), including sinusitis, acute otitis, bronchitis, bronchiectasis, and pneumonia. The second common complications were infections of skin and mucous membranes. There were 38 cases of skin and mucous membrane infection (33.9%), including 11 cases of mouth ulcers, 9 cases of perianal abscess, 7 cases of mycotic stomatitis, 7 cases of skin pustules and nodules, and 4 case of eczema. The third common complication was digestive tract infection, of which diarrhea was found in 26 cases (23.2%). However, diarrhea not only is caused by infection, but also caused by immune disorder. In addition, we found 7 cases with infection of central nervous system (CNS). It should be noted that 23 children (20.5%) had adverse event following immunization (AEFI), involving Bacillus Calmette-Guérin (BCG)-osis, local skin infection, and fever. Furthermore, 21 patients (17%) had BCG-osis after BCG vaccination, and BCG-osis was mainly observed in patients with SCID (9 cases), chronic granulomatous disease (7 cases), idiopathic primary hypogammaglobulinemia (4 cases), and interferon-gamma receptor deficiency (1 case). Thus, AEFI may lead to the consideration of the possibility of PID. Additionally, a variety of clinical manifestations, such as leukopenia, anemia, thrombocytopenia, seizure, rash, and lymphoma, were rarely observed in the patients with PID in our study.

**Table 3:** Clinical manifestations and complications of patients with primary immunodeficiency diseases

Type	Respiratory infections	Repeated Diarrhea	Bacterial Infection Of Skin and Mucous Membrane	Fungal Infection	Meningitis	BCG-osis
SCID	19	13	8	6	4	9
HIGM	26	6	10	7	0	4
WAS	5	2	4	0	0	0
DGS	1	0	0	0	0	0
DKC	2	1	1	0	0	0
EDA-ID	1	0	0	0	0	0
HIES	1	0	0	0	0	0
Severe reduction in all serum immunologlobulin isotypes with profoundly decreased or absent B cells	14	1	2	1	2	0
CVID	3	0	0	0	1	0
APDS	1	0	0	0	0	0
IPEX syndrome	1	2	1	1	0	0
Immune dysregulation with colitis	0	1	1	0	0	0
CHS	0	0	1	0	0	0
Congenital neutropenias	7	0	1	2	1	0
CGD	6	1	6	0	0	7
IFN-γ receptor deficiency	1	0	1	0	0	1
CARD9 deficiency	0	0	1	1	1	0
Complement deficiencie	1	0	1	0	1	0
Total (%)	89 (79.5)	26 (23.2)	38 (33.9)	18 (16.1)	10 (8.9)	21 (18.8)

#### Outcomes

Of the 112 patients, 43 (33 males and 10 females, 38.4%) died due to recurrent and severe infections during the follow-up. The mean age of death for SCID patients was 7 months, while patients with HIGM syndrome and predominantly antibody deficiencies had the mean age of death equal to 88.5 and 46 months, respectively (Table 2). Of these, 38 died during hospitalization, and 5 died after discontinuing treatment (all with SCID). In addition, 65 patients survived, and 4 patients were lost follow-up during the follow-up period. Respiratory failure after pneumonia, sepsis, complications after allogeneic hematopoietic stem cell transplantation (allo-HSCT), disseminated BCG, and pericardial effusion were the most common causes of death as well. It is noteworthy that 93.8% (30/32) of the SCID patients died (including 6 cases who underwent allo-HSCT) except for 1 case who underwent allo-HSCT and the other who is currently alive and preparing to undergo allo-HSCT. In the present study, a total of 47 cases underwent allo-HSCT, and the 2-year overall survival (OS) rate was 78.7%. The OS widely differed among PID patients with different phenotypes who underwent allo-HSCT (Table 4). Among 7 patients with SCID who underwent allo-HSCT, 3 patients died of sepsis, 2 patients died of graft-versus-host-disease (GVHD), and 1 patient died of pretreatment before allo-HSCT, while only 1 case was survived. Among 24 patients with HIGM syndrome who underwent allo-HSCT, 3 patients died of sepsis, 1 patient died of GVHD, and 20 cases were survived. The remaining cases were all survived and exhibited complete immune reconstitution. The 2-year OS rate for patients with SCID, HIGM syndrome, and the remaining patients with PID who received allo-HSCT was 14.3, 83.3, and 100%, respectively (Table 4).

**Table 4:** The overall survival of PIDs patients after allogeneic HSCT

Type	No. of Cases	No. of Survival	No. of Death
Total	47	37	10
SCID	7	1	6
HIGM	24	20	4
WAS	9	9	0
DKC	1	1	0
IPEX syndrome	2	2	0
CHS	1	1	0
Congenital neutropenias	1	1	0
CGD	1	1	0
IFN-γ receptor deficiency	1	1	0

## Discussion

PIDs are rare inherited diseases involved the immune system, typically associating with recurrent and severe infection, autoimmune disease and increased incidences of malignancies. At present, fast diagnosis of PID in non-specialized hospitals or clinics across China accompanies with great challenges. Delayed diagnosis and misdiagnosis commonly occur, mainly leading to poor clinical prognosis. In the current research, we studied 112 patients with PID during 6 years in our hospital based on their clinical, immunological, and molecular characteristics, aiming to provide a reference for perfect diagnosis of PID.

In the present research study, the most common PID was combined immunodeficiencies (28.6%), followed by HIGM syndrome (24.1%), predominantly antibody-deficiency diseases (17.8%), combined immunodeficiencies with associated or syndromic features (12.5%), congenital defects of phagocyte number or function (10.7%), diseases of immune dysregulation (3.6%), defects in intrinsic and innate immunity (1.8%), and complement deficiencies accounted for about 0.9% of cases, which were found to be different from those previously reported [6–8] probably because our hospital is a tertiary referral center and our cases were in-patients and a number of them were admitted from elsewhere. Therefore, the proportion of patients with PID, who are prone to have severe complications and difficult to diagnose, significantly increased. In addition, our hospital is one of the main referral centers for allo-HSCT in China, and some of the PID patients in our study were previously diagnosed with PID in other hospitals who needed to receive allo-HSCT. Furthermore, we cannot ignore a possibility that a number of differences were based on race and relative incidence of disease in our population. Our data further showed that the male/female ratio was 6.0:1, and 60.7% of the cases presented symptoms less than 6 months. Besides, 17.8% of the patients in our study had family history of PID, which was found similar to a previous study [8].

Repeated and chronic infections, particularly pulmonary infections, are a main feature of PID. Large sample cohort studies on PID indicated that almost all patients with PID had a history of recurrent infection before diagnosis was finalized [9–11]. In agreement with those reports, the present study revealed that 97.3% of PID patients presented recurrent infections before diagnosis. The most common occurrences were respiratory tract infection, followed by bacterial infection of the skin and mucous membranes in 38 cases (33.9%). Of note, gastrointestinal disorders are frequent in patients with PID [12, 13]. Moreover, 26 (112, 23.2%) patients with infectious or noninfectious diarrhea showed a poor growth as well. Besides, PID-related gastrointestinal diseases not only are caused by infection, but also by autoimmunity, an inflammatory response, or malignancy. Recurrent gastrointestinal symptom could be the first presentation of PID, thus physicians should be aware of the possibility of PID in patient with intractable diarrhea, malabsorption, and failure to thrive, especially those cases who failed to respond to conventional treatment strategies [14, 15].

Sarmiento et al. reported 7.65% of AEFI in patients with PID [16]. The majority of cases of AEFI have occurred in patients with CGD, SCID, and idiopathic primary hypogammaglobulinemia. It was reported that BCG is a vaccine, mainly associating with AEFI in patients with PID [17]. In the current study, AEFI was found in 20.5% of patients with PID after vaccination, especially with BCG vaccine, and included extra regional lymph nodes, skin, or lungs as the most common clinical presentations. For patients with CGD and SCID, the percentage of BCG-osis was 100 and 28.1%, respectively, which found to be in line with previously reported findings [18–20]. Notably, 17.8% of the cases included family history of PID. Because BCG vaccination is routinely carried out at birth in China, PID patients who receive BCG vaccination before immune deficiency are highly suspected. BCG vaccination should be avoided if any family history or clinical or laboratory evidence concerns a neonate’s immune competency. Moreover, disseminated BCG infection should be suspected in any vaccinated infants who accompany with a persistent fever or comparable disease of unknown etiology.

In addition to recurrent and severe infections, immunity disorder is commonly observed in PID patients, especially in patients who accompany with predominantly antibody deficiencies [21, 22]. A recent study, that involved an Iranian cohort of 471 patients, reported inflammatory manifestations in 26.5% of patients. Furthermore, the prevalence of immunity disorder appeared to increase with age in PID cohorts, influencing a significant proportion of patients [23]. In this study, we also found that not only autoimmune gastrointestinal disease, but also autoimmune cytopenias were the common autoimmune manifestations in patients with PID. When a child associates with autoimmune manifestations, the possibility of incidence of PID should be highly taken into account.

Immunoglobulin replacement therapy and allo-HSCT are effective therapeutic strategies for patients with PID. On the basis of previous reports, immunoglobulin replacement therapy was found to be effective for 83% of children with hypogammaglobulinemia [24, 25]. At present, the majority of patients with PID only receive symptomatic treatment. Allo-HSCT, which has been used as an effective treatment for PID [26, 27], isn’t frequent in China. The 2-year OS rate in the SCID patients was 90% during 25 months of follow-up [28]. A total of 47 cases underwent allo-HSCT and the OS rate was 78.7% (37/47) in the present study, which was in agreement with those findings previously reported [27]. However, the OS widely differed among PID patients with different phenotypes who underwent allo-HSCT. The 2-year OS rate for SCID, HIGM syndrome, and the remaining of PID patients who underwent allo-HSCT was 14.3, 83.3, and 100%, respectively. The OS of SCID is mainly poor because SCID patients are often severely infected, and bone marrow transplantation of severely infected SCID children has a poor prognosis. Compared with patients with active infection and older age who underwent allo-HSCT, OS was better in those patients who received a transplant when they were younger and free from infection. Thus, allo-HSCT is more effective in younger PID children, and allo-HSCT is often helpful when an appropriate donor is available.

However, the present study contains some limitations, including its retrospective nature, small sample size, and all the cases were recruited at a single center. A possible selection bias also might be present because the clinical data were taken from hospitalized children with PID. Further researches involving more PID patients and multiple centers are required in the future study.

## Conclusions

Our study indicated that PID typically emerges at early age. Recurrent infection and serious infection were the most common clinical manifestations. Delayed diagnosis or misdiagnosis may lead to poor clinical prognosis as well. Allo-HSCT is a relatively effective therapeutic strategy for PID patients. Our study may provide a reliable reference for pediatricians to diagnosis children with PID.

## Data Availability

The datasets used in the current study are available from the corresponding author upon reasonable request.

## Abbreviations

AEFI: adverse events following immunization
Allo-HSCT: allogeneic hematopoietic stem cell transplantation
APDS: activated PI3Kδ syndrome immunodeficiency
BCG: Bacillus Calmette–Guérin
CGD: chronic granulomatous disease
CHS: Chediak-Higashi syndrome
CVID: common variable immunodeficiency disorders
DGS: DiGeorge syndrome
DKC: dyskeratosis congenital
EDA-ID: anhidrotic ectodermodysplasia with immunodeficiency
GVHD: graft versus host disease
HIES: Hyper-IgE syndromes
HIGM: hyper-IgM syndrome
IPEX: immunodysregulation, polyendocrinopathy, enteropathy X-linked syndrome
IUIS: International Union of Immunological Societies
OS: overall survival
PIDs: primary immunodeficiency diseases
SCID: severe combined immune deficiency
WAS: Wiskott–Aldrich syndrome
XLA: X-linked agammaglobulinemia
